# Optimal Cardiac Resynchronization Therapy Pacing Rate in Non-Ischemic Heart Failure Patients: A Randomized Crossover Pilot Trial

**DOI:** 10.1371/journal.pone.0138124

**Published:** 2015-09-18

**Authors:** Adam Ali Ghotbi, Mikael Sander, Lars Køber, Berit Th. Philbert, Finn Gustafsson, Christoffer Hagemann, Andreas Kjær, Peter K. Jacobsen

**Affiliations:** 1 The Heart Center, Department of Cardiology, Rigshospitalet Copenhagen University Hospital, Copenhagen, Denmark; 2 Department of Clinical Physiology, Nuclear Medicine & PET, Rigshospitalet Copenhagen University Hospital, Copenhagen, Denmark; Kurume University School of Medicine, JAPAN

## Abstract

**Background:**

The optimal pacing rate during cardiac resynchronization therapy (CRT) is unknown. Therefore, we investigated the impact of changing basal pacing frequencies on autonomic nerve function, cardiopulmonary exercise capacity and self-perceived quality of life (QoL).

**Methods:**

Twelve CRT patients with non-ischemic heart failure (NYHA class II–III) were enrolled in a randomized, double-blind, crossover trial, in which the basal pacing rate was set at DDD-60 and DDD-80 for 3 months (DDD-R for 2 patients). At baseline, 3 months and 6 months, we assessed sympathetic nerve activity by microneurography (MSNA), peak oxygen consumption (pVO_2_), N-terminal pro-brain natriuretic peptide (p-NT-proBNP), echocardiography and QoL.

**Results:**

DDD-80 pacing for 3 months increased the mean heart rate from 77.3 to 86.1 (p = 0.001) and reduced sympathetic activity compared to DDD-60 (51±14 bursts/100 cardiac cycles vs. 64±14 bursts/100 cardiac cycles, p<0.05). The mean pVO_2_ increased non-significantly from 15.6±6 mL/min/kg during DDD-60 to 16.7±6 mL/min/kg during DDD-80, and p-NT-proBNP remained unchanged. The QoL score indicated that DDD-60 was better tolerated.

**Conclusion:**

In CRT patients with non-ischemic heart failure, 3 months of DDD-80 pacing decreased sympathetic outflow (burst incidence only) compared to DDD-60 pacing. However, Qol scores were better during the lower pacing rate. Further and larger scale investigations are indicated.

**Trial Registration:**

ClinicalTrials.gov NCT02258061

## Introduction

Cardiac resynchronization therapy (CRT) has become an important treatment strategy for a select group of heart failure (HF) patients with electrical dyssynchrony, and several studies have documented the beneficial effects of CRT on mortality and morbidity in such patients. [1, 2] The prevalence of HF is projected to increase by 25% by 2030 compared to 2013 [3], and approximately 10% of an unselected group of HF patients could be eligible for biventricular pacing (BiV), [4] thus indicating a likely future increase in CRT utilization.

Although the majority of correctly selected patients respond favorably to CRT, 25–30% show little or no improvement after device implantation. To increase response rates, resources have focused on programming optimization, particularly atrioventricular (AV) and interventricular (VV) timing intervals. [5–7] However, few studies have examined the optimal basal atrial pacing rate and its impact on long-term outcome in CRT patients. Increasing pacing rates in BiV mode have demonstrated positive acute hemodynamic effects (e.g., decreased filling pressure and increased cardiac output). [6, 8–10] Furthermore, an increased atrial basal pacing rate and HR (heart rate) could prove to be favorable in HF patients with chronotropic incompetence (attenuated HR response to exercise),[11, 12] which is associated with increased cardiac and all-cause mortality. [13, 14] In contrast, beta-blocking therapy appears to be associated with better outcomes in terms of death and transplantation in CRT patients,[15] which aligns with the beneficial effect of a lower HR in HF patients without CRT. [16] Moreover, the “Dual Chamber and VVI Implantable Defibrillator Trial”[17] tested whether the dual-chamber pacing (DDDR-70) mode could improve hemodynamics and reduce major cardiac adverse events (MACE) compared to ventricular back-up pacing (VVI-40). Surprisingly, the trial showed an increase in the composite endpoint in the pacing group; however, the results were not directly comparable with the CRT population due to the lack of left ventricular pacing. Therefore, the optimal basal atrial pacing rate in CRT patients remains poorly defined.

The aim of this randomized, double-blind, crossover pilot trial was to investigate the impact of 60-bpm (DDD-60) to 80-bpm (DDD-80) basal atrial pacing rates on autonomic nerve function in CRT patients, as assessed by (i) microneurography and NT-proBNP, (ii) cardiopulmonary exercise tests and (iii) self-perceived quality of life (QoL).

## Methods

### Study population

Fifteen patients were included in the study from the 18^th^ of August 2011—5^th^ of January 2013; 3 patients withdrew (1 due to throat cancer, 2 due to long commuting distances), leaving 12 patients with idiopathic dilated cardiomyopathy who had had a CRT device implanted (the initial indications were in accordance with European guidelines) [18] at least 6 months prior to study inclusion. The steering committee decided to restrict inclusion to non-ischemic CRT patients to assess any potential risk before decision on inclusion of ischemic CRT patients. The patients were in New York Heart Association (NYHA) functional class II (n = 7) or III (n = 5), with mean a left ventricular ejection fraction (LVEF) of 38%±10. The patients were required to be in sinus rhythm and hemodynamically stable as well as to demonstrate biventricular pacing > 90% of the time and to be undergoing optimal medical treatment for HF, with no changes in medications during the past 3 months. All patients were considered to be CRT responders based on either NYHA or LVEF improvements. Patients were excluded if they were NYHA I, unable to perform the exercise test (e.g., diagnosed with chronic obstructive pulmonary disease, severe arthritis), diagnosed with cancer, had plasma creatinine levels > 200 micromoles per liter, or were admitted for decompensated HF or acute coronary syndrome in the preceding 3 months.

### Ethics

All participating patients provided informed written consent before study participation. All relevant approvals were obtained from Capital Region of Denmark ethical committees (H-3-2011-034) the 16^th^ of May 2011, and the study was performed in compliance with the Declaration of Helsinki. The study was not registered in a public database before patient enrollment due to its nature as a pilot study. The authors confirm that all ongoing and related trials for this intervention are registered.

### Study protocol

**Fig 1:**
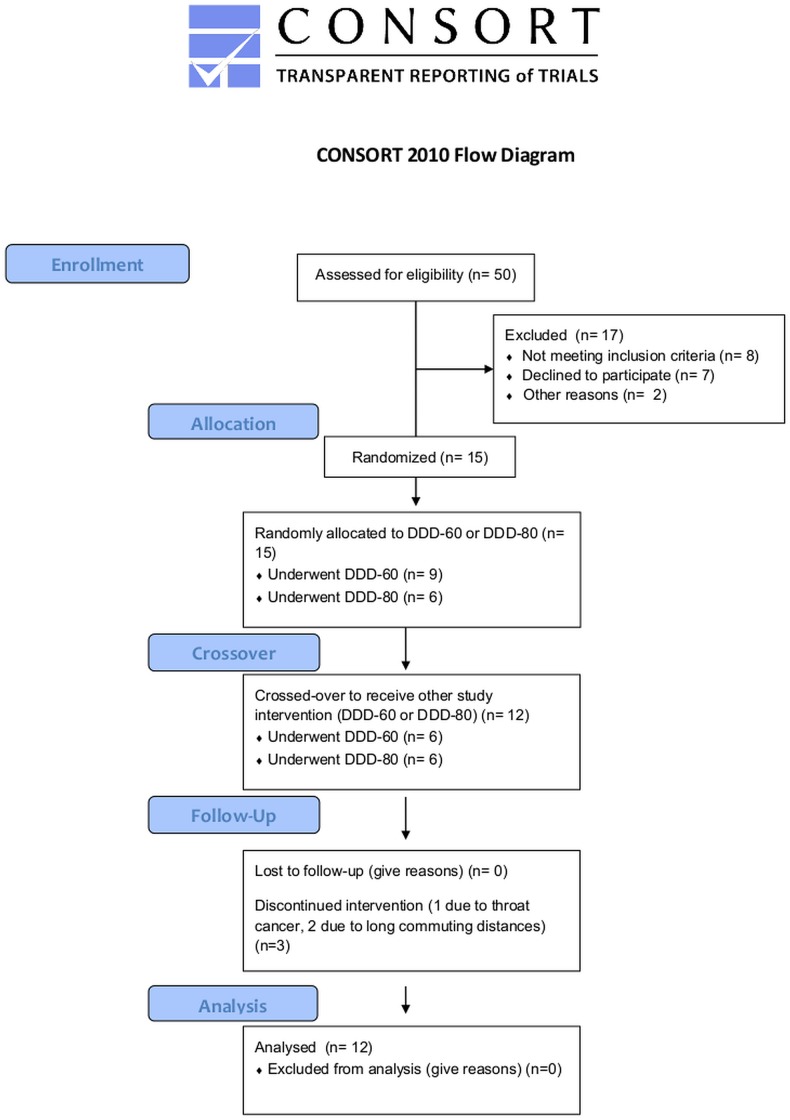
Study design. Cross-over design, including baseline and 3- and 6-month follow-up. QoL: quality of life questionnaire; MNSA: muscle sympathetic nerve activity; CRT: cardiac resynchronization therapy; bpm: beats per minute.

This double-blind, randomized, crossover pilot study tested two basal atrial pacing rates: DDD-60 versus DDD-80. Each patient was paced for 2 90-day periods, with DDD-60 and DDD-80 in a randomly assigned sequence. Complete patient histories were obtained, and physical examinations were performed for all patients at baseline, 3 months after phase 1, and 6 months after phase 2; *see* Fig 1 *for the study protocol*.

After concluding each phase, the patients underwent echocardiography, and blood samples were taken. In addition, the patients completed two separate self-perceived QoL questionnaires and performed cardiopulmonary bicycle tests; sympathetic nerve activity was assessed using microneurography (MSNA), and the CRT device was interrogated and finally reprogrammed to the opposite pacing rate or to baseline settings at the final visit. MSNA and bicycle tests were performed on two separate days at each phase to avoid exercise-induced elevated sympathetic activity. The CRT settings were blinded to the attending physician, and the patients were unaware of the study assignment sequence.

### QoL questionnaire

HF patients are compromised by an array of symptoms, including dyspnea, limited physical activity, and fatigue. Furthermore, increased medication intake and the possible side effects of those medications, as well as the progressive loss of self-reliance and recurrent hospitalization, all affect and impact QoL. [19] We administered two distinct questionnaires (the Minnesota Living with HF [LIfHE] questionnaire and Medical Outcome Survey Short Form SF-36]) [20] to fully comprehend changes in the signs and symptoms of HF, physical and sexual activity, and psychological and socioeconomic conditions. LIfHE is a well-validated and popular tool used to assess QoL in HF patients; it contains 21 questions designed to measure the above-mentioned factors. [21] SF-36 is comprised of 36 items, which are subdivided into 8 health dimensions (vitality, physical functioning, bodily pain, general health perceptions, physical role functioning, emotional role functioning, social role functioning, mental health). The scores are transformed into a 0–100 scale, with each question equally weighted. High scores indicate less restraint, and lower scores indicate more restraint.

### Device settings and programming

In this study, the time between implantation of the CRT device and randomization averaged 20.8 ± 10.7 months. Eleven patients had St. Jude devices, and one patient had a Medtronic device. The majority of patients were in DDD (n = 10) and DDD-R (n = 2) mode. Two highly experienced technicians managed the device settings and ensured optimal programming, but they were not otherwise involved in this study. The device was randomly programmed to a basal pacing rate of 60 bmp in the first phase and 80 bmp in the second phase or vice versa. Atrioventricular and interventricular delays were programmed according to the local electrophysiologist’s discretion, and these settings were not changed during the study.

Device transcripts were obtained at each phase to register the levels of atrial and ventricular sensing, pacing, BiV pacing (%), and HR histograms.

### Exercise protocol

A symptom-limiting bicycle (Ergoline, Baden-Württemberg, Germany) exercise test was performed to measure maximum work capacity and oxygen consumption at each phase. The HR and 12-lead electrocardiogram were recorded continuously, and blood pressure was recorded every 2 minutes. The initial workload was set to 25 Watts (W) and increased by 25 W every 2 minutes until exhaustion, at a rate of 60–70 revolutions per minute. During the test, the patients inspired room air through a low-resistance mouthpiece, with the nostrils tightly blocked. The expired oxygen and carbon dioxide partial pressures were measured (using the breath-by-breath method) with a gas analyzer (Innocor, Innovision Aps, Glamsbjerg, Denmark). Calibration procedures were performed according to the manufacturer’s specification immediately before the tests, and the following parameters were measured: peak oxygen consumption (pVO_2_), which was defined as the highest VO_2_ (averaged over a 20-30-second period) achieved at maximal effort; respiratory exchange ratio (peak VCO_2_/VO_2_ = RER); peak minute ventilation—carbon dioxide relationship (pVE/VCO_2_ slope), which was derived using least-squares linear regression (y = mx + b; m = slope) after entering the data into a spreadsheet (Excel; Microsoft); [22] and maximal workload (Watt_max_), which was defined as the highest workload maintained for a minimum of 45 seconds.

### Muscle Sympathetic Nerve Activity measured with microneurography

Resting muscle sympathetic nerve activity (MSNA) recordings were obtained for all patients at both pacing rates; all patients were studied using the same set-up and were placed in the semi-recumbent position after they had rested for a minimum of 30 minutes. Tungsten microelectrodes (Iowa Bioengineering) were inserted into the common peroneal nerve just below the knee (typically the left peroneal nerve). The correct position of the microelectrode tip is within close proximity to the post-ganglionic sympathetic nerve fiber bundles that innervate the vascular smooth muscle of the peroneal muscle or anterior tibial muscle vascular beds. These bundles are typically located close to the motor neurons innervating the same muscle group and close to the afferent nerves from these muscle groups. Intraneural stimulation was used to locate the microelectrode position by searching for muscle twitching and by testing muscle activation by tapping the muscle belly to engage the muscle stretch receptors. The firing of the sympathetic fibers was then recorded in the resting leg by pre-amplifying the signals (x1000) and by filtering (bandwidth 700–2000 Hz, half cut-off filters), rectifying and integrating the signals with a time-constant of 0.1 second and then further amplifying the signals (x95.5) (Iowa Bioengineering). The resulting integrated neurograms were then studied, and only the recordings with bursts that reached a signal:noise ratio of at least 3:1 were accepted for analysis. A minimum of 10 minutes was allowed for the recording and for the patient to settle after manipulating the needle; the subsequent 10 minutes of MSNA were used for the analysis. All data were analog-digital converted and saved for later analysis (ADInstruments, LabChart 7.0). The analysis was performed using macro-programming in LabChart in a manner in which all cardiac cycles were tested for significant sympathetic activity, and sympathetic bursts were counted if the amplitude and area under the curve (method: Integral relative to Baseline) [23] reached certain predetermined levels that were normalized to the noise level. The validity of these semi-automated analyses were tested by an experienced microneurographer (MS) who was blinded to the macro-result and who compared the results to manual scoring; on average, differences of less than 10% were found for the 6 conditions (3 patients) tested.

MSNA bursts are strictly synchronized to the cardiac cycle by the arterial baroreflex. Thus, arterial baroreceptors are unloaded to the extent to which they allow sympathetic outflow from the brainstem only during diastole. For every heart beat (and every diastole), only one integrated sympathetic burst can occur, but MSNA bursts do not occur with all heart beats (the cycles in which the diastolic pressure is too high to unload the baroreceptors are not followed by a sympathetic burst). In the present study, we manipulated the HR and therefore also manipulated the maximum possible MSNA burst frequency. For this reason, the MSNA burst frequency (bursts/minute) and the MSNA burst incidence were analyzed after being normalized to the HR (bursts/100 RR).

### Echocardiography

Standard echocardiography was performed according to the European Association of Echocardiography guidelines. [24] The LVEF, left ventricular end diastolic dimension (LVEDD), and end systolic dimensions (LVEDS) and tricuspid annular plane systolic excursion (TAPSE) were measured.

### Blood samples

NT-proBNP plasma levels were analyzed at baseline and at the end of each phase using electrochemiluminescence immunoassays. The patients were allowed to rest for 15 minutes before the samples were taken.

### Study endpoints

The primary study endpoints were changes in NT-proBNP and sympathetic activity, as assessed by MSNA. Additional endpoints were exercise capacity in the form of pVO_2_ and pVE/VCO_2_ as well as self-perceived QoL.

## Safety

An adjudication committee oversaw the study. If the baseline NT-proBNP increased more than 50% or if the NYHA class deteriorated in more than 5 patients during the fast-pacing period, the study would have been terminated early.

## Statistical Analysis

Continuous variables are presented as the means ± standard deviations (SDs), and categorical variables are presented as frequencies or percentages (%). All variables were tested for normality using the Kolmogorov-Smirnov test and normality plots. NT-proBNP underwent logarithmic transformation to obtain a normal distribution.

To rule out carryover and time effects in a crossover trial, the sum of the variables measured in the two periods were calculated for each subject and compared to the two sequence group means using an unpaired t-test. [25] There are arguments for [26, 27] and against [28] the use of baseline measurements in crossover designs; therefore, a two-way repeated measures analysis of variance (ANOVA) of baseline values and a paired Student’s t-test (excluding baseline values when comparing the two pacing rates for endpoints) were performed. Furthermore, a sensitivity analysis comparing baseline MSNA values to DDD-60 study period was performed, along with an intra-class correlation to estimate the MSNA variability. Wilcoxon signed-rank tests were used for non-parametric statistics, as appropriate. To enhance the understanding of the magnitude of an observed effect, we calculated the effect size (parametric r = $\sqrt{\frac{t^{2}}{t^{2}+df}}$, *t* ^*2*^ = *t-score*, *df = degrees of freedom*; non-parametric r = $\frac{z}{\sqrt{N}}$, *z* = *z-score*, *N = number of total observations*), as suggested by Fritz et al. al. [29], and *r = 0*.*10*, *r = 0*.*3*, and *r = 0*.*5* constituted small, medium, and large effects, respectively. A P value of <0.05 was considered significant. All statistical analyses were performed using SPSS® (version 19) statistical software (Chicago, IL, USA).

### Power calculations

These estimates are linked with high uncertainties since few studies have conducted MSNA measurements on CRT patients (hence, this pilot trial). Previous studies [30, 31] show a standard deviation for MSNA of 9 bursts/min and 12 bursts/100RR, and a relevant difference of 20 and 14, respectively. Sample size calculation is based on the paired difference model. With a power (1-beta) of 80% and a two-sided alpha of 5%, the null hypothesis can be rejected with a paired sample size of 8. [32]

## Results

**Fig 2:**
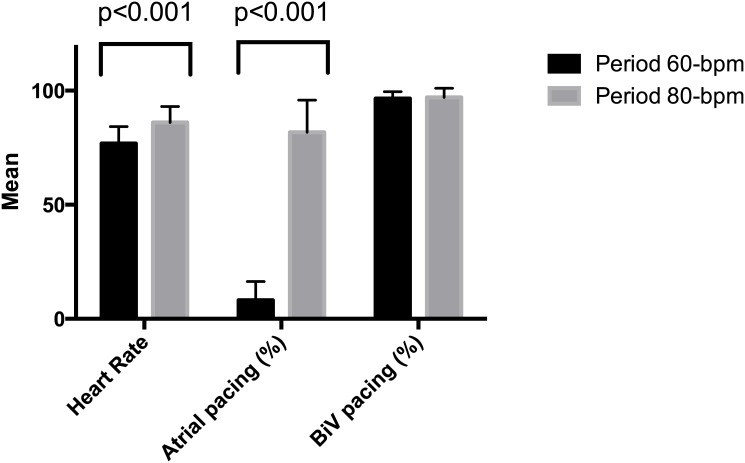
Mean heart rate, atrial pacing and biventricular (Biv) pacing (%) in periods 60 bpm vs. 80 bpm.

The baseline characteristics of the patients are shown in Table 1. The cohort comprised 6 males and 6 females, the mean patient age was 66 ± 9 years, and BiV pacing was present in 97±4% of the patients. All patients were receiving beta-blocker and either an angiotensin-converting enzyme inhibitor or an angiotensin receptor blocker. The pre-tests showed no carry-over or time effects for the endpoints in the sequence groups. There were no significant changes in the levels of serum hemoglobin, potassium, sodium or creatinine during the study, and the data from the ventricular histograms revealed a significant difference in the mean HR between DDD-60 versus DDD-80 pacing (the mean difference was 8.7 ± 6 bmp, p = 0.001). The atrial pacing level was elevated during the 80-bpm period compared to the 60-bpm period (82±14% vs. 8±8%, p = 0.001), Fig 2.

**Table 1:** Baseline characteristics.

Baseline Characteristics	
Participants	N = 12
Age (years): mean ± SD	66±8.8
Sex (N and % female)	6 (50%)
Etiology: DCM	12 (100%)
NYHA:
II	7 (58%)
III	5 (42%)
Rhythm: SR	12(100%)
CRT duration (months)	21±11
CRT mode:
DDD	10 (83%)
DDDR	2 (17%)
Medication:
BB	12 (100%)
DIU	11 (92%)
ACE/ARB	12 (100%)
SPIR	4 (33%)
Pre-CRT LVEF (%)	27±5.6
Post-CRT LVEF (%)	38±10

DCM: idiopathic dilated cardiomyopathy; NYHA: New York Heart Association Functional Class; SR: sinus rhythm; BB: beta-blockers; DIU: diuretics; ACE/ARB: angiotensin-converting enzyme inhibitor or angiotensin receptor blocker; SPIR: spironolactone; EF: left ventricular ejection fraction; CRT: cardiac resynchronization therapy.

**Fig 3:**
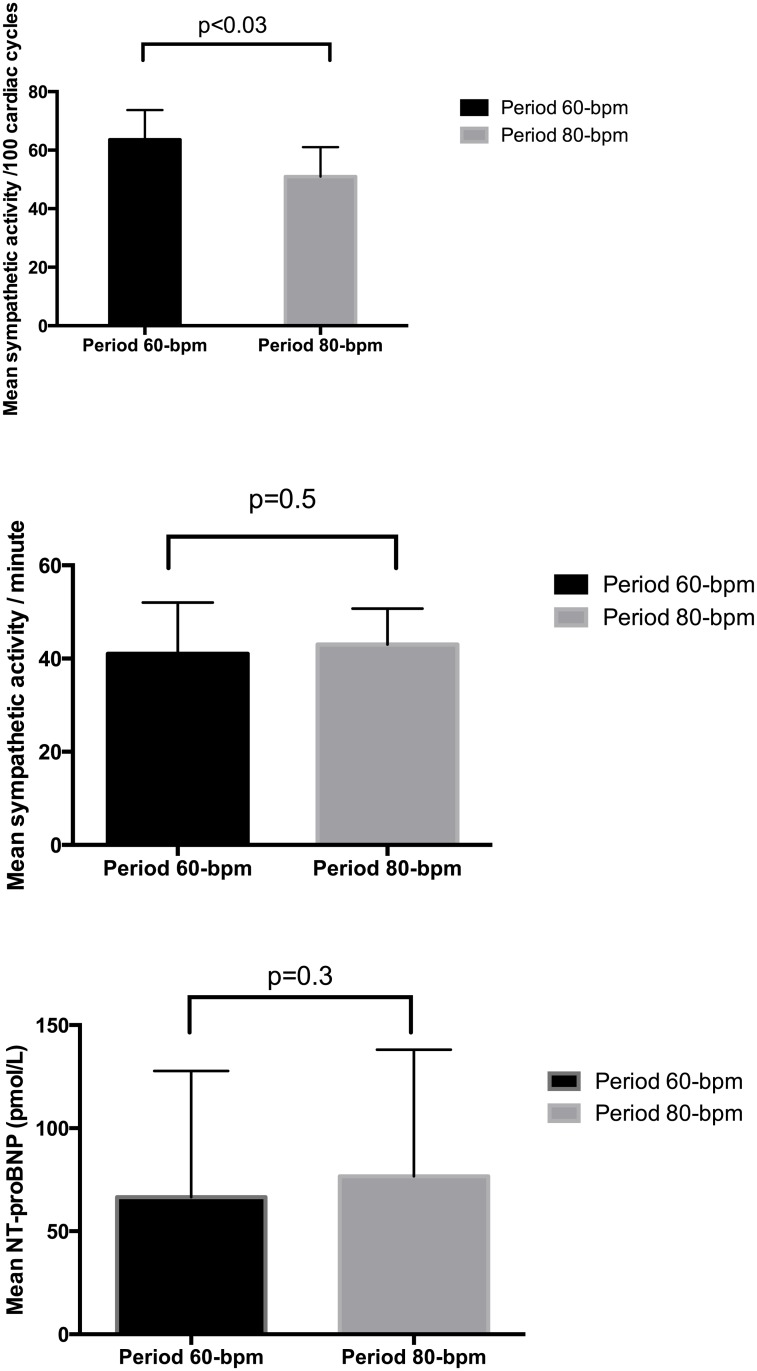
Top panel: Sympathetic activity in the 60-bpm vs. 80-bpm period. Bursts/100 RR: sympathetic bursts per 100 cardiac cycles. Lower panel: Mean NT-proBNP (pmol/L) in the 60-bpm vs. 80-bpm period.

The crude median NT-proBNP after the DDD-60 study period was 59 pmol/L [interquartile range (IQ), 12.9–106.8] compared to 75 pmol/L [IQ, 13.1–132–8] after the DDD-80 study period. However, no significant difference in log-NT-proBNP was observed between the two pacing rates by either the paired t-test (p = 0.3) or ANOVA (p = 0.44), Fig 3.

**Fig 4:**
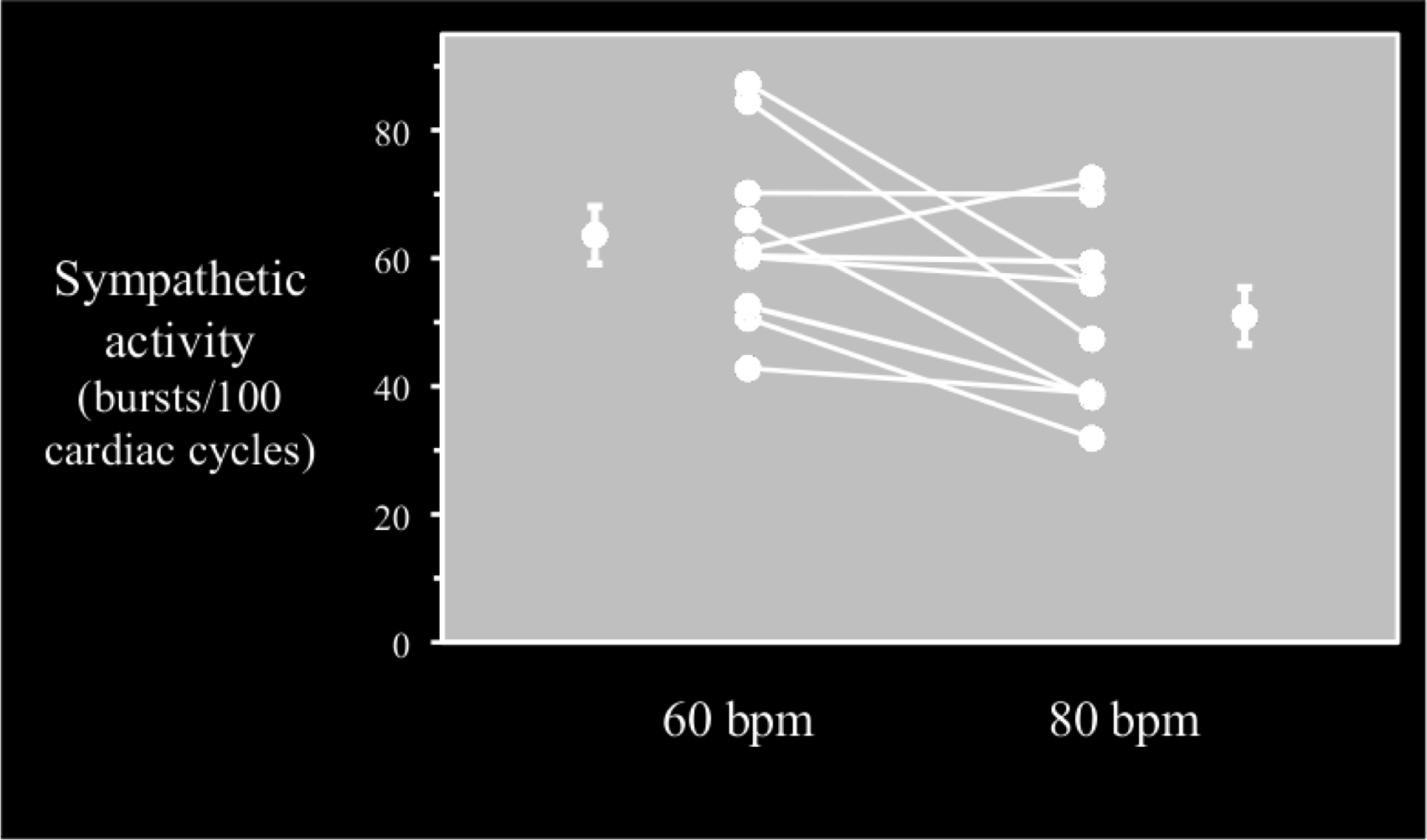
Sympathetic activity at individual levels during the 60-bpm and 80-bpm test periods. Bursts/100 RR: sympathetic bursts per 100 cardiac cycles during the DDD-60 and DDD-80 periods.

**Fig 5:**
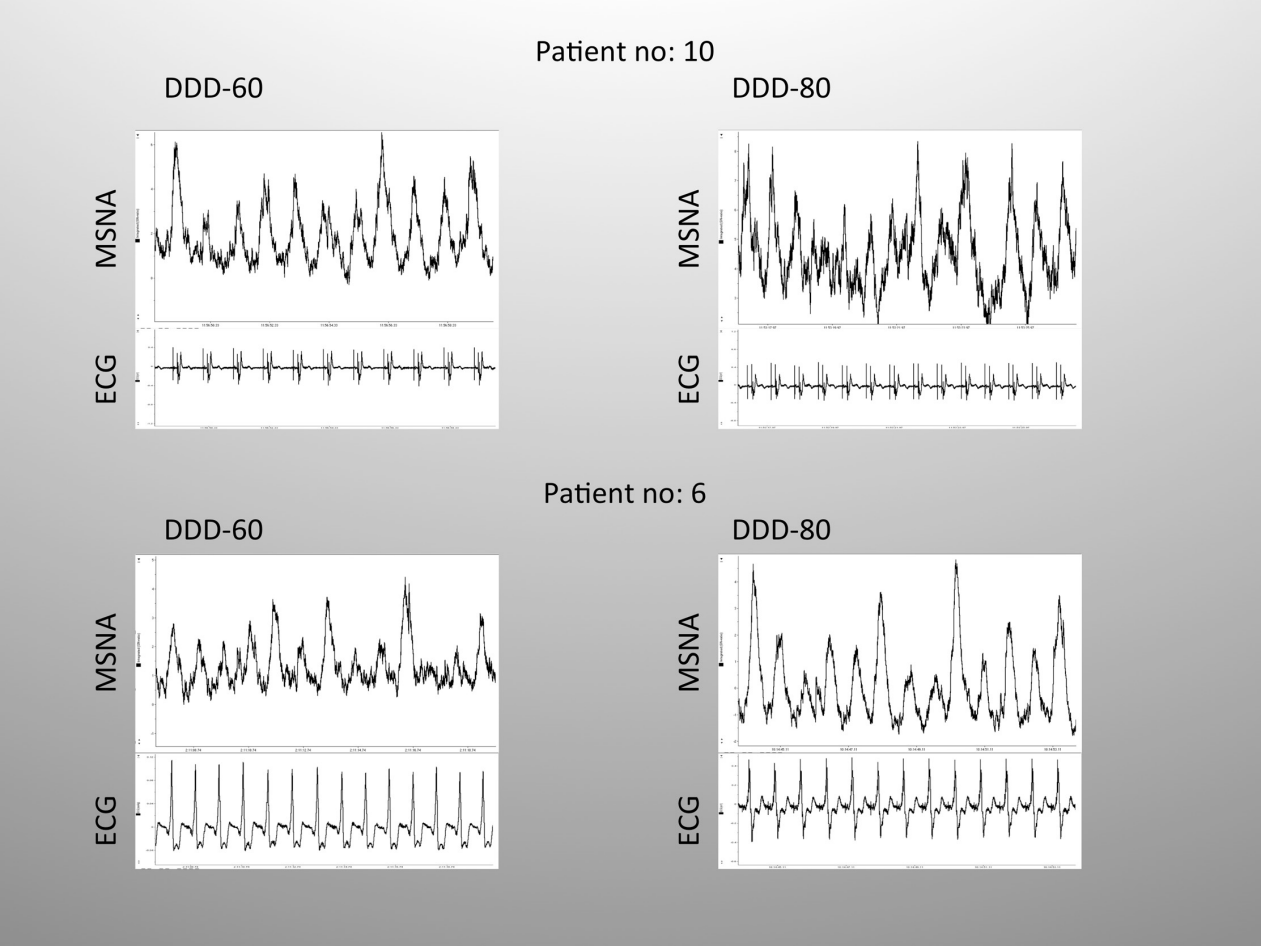
Examples of sympathetic activity and ECG recording. Mean voltage neurograms for MSNA and electrocardiograms (ECG) in two patients. Patient no. 10 (upper panel) had a significant decrease in sympathetic activity during the DDD-80 period, while patient no. 6 (lower panel) experienced an increase in sympathetic activity during the DDD-80 period compared to the DDD-60 period.

Sympathetic activities were available for 10 patients (it was not possible to locate the peroneal nerve in 2 patients), and they dropped significantly during the DDD-80 study period compared to the DDD-60 period (51±14 bursts/100 RR vs. 64±14 bursts/100 RR, respectively; p<0.05; effect size, r = 0.73). With the exception of one patient (no. 6), all patients experienced decreased sympathetic activity during the DDD-80 study period compared to the DDD-60 study period. Between the two study periods, there was no significant change in the bursts per minute (41±11 burst/min in DDD-80 period vs. 43±7.7 burst/min in the DDD-60 period, p>0.52). A sensitivity analysis was performed where baseline measurements were compared to DDD-60 period (only patients with DDD/DDD-R = 60 at baseline, n = 9). Baseline MSNA values (57 burst/100 RR) were not significantly different from the DDD-60 period, p>0.11 (results not shown). A high degree of reliability was found between the two measurements. The average intra-class correlation was 0.781 (CI:0.144–0.949, p<0.01), Figs 4–5.

All patients completed the exercise tests, and they were limited due to breathlessness or fatigue. The majority of participants did not achieve RER>1.0; and at both pacing rates, the mean RER equaled 0.94±0.11. After the DDD-80 study period, the mean pVO_2_ increased non-significantly to 16.7±6 mL/min/kg, compared with 15.6±6 mL/min/kg after the DDD-60 study period (p = 0.43). The pVE/CO_2_ slope dropped to 30.2±5.9, at 80 bpm, from 32.3±6.1, at 60 bpm, (p = 0.17). The maximal workload marginally improved in the 80-bpm study period (88±31 W vs. 85±40 W during the 60-bpm period, p = 0.83), and the exercise duration remained unchanged during the study.

The SF-36 Health Survey and LIhFE were completed by all patients (except for one patient in the 80-bpm study period) to assess QoL. SF-36, which included the 8 dimensions of health perception, revealed a significant difference in the “Bodily Pain” score (a high score indicates the absence of pain): 67±23 in the 60-bpm study period versus 59±30 in the 80-bpm study period (p<0.03, *r* = 0.45). Furthermore, the component score of Mental Health was significantly higher in the 60-bpm study period compared to the 80-bpm study period (53±11 vs. 49±11, respectively, p<0.04, *r* = 0.43). There were no significant differences in the scores of the dimensions of health perception or physical health. The LIHFE survey did not reveal any significant differences.

No significant changes were observed in the echocardiography or blood pressure measurements (systolic, diastolic, and mean arterial pressure) between the two study periods.

## Discussion

The present pilot trial examined the effects of a basal pacing rate of 80 bpm compared to 60 bpm in CRT patients in an array of physiological and biometrical parameters. We found no beneficial or detrimental effects of 80 bpm in terms of the NT-proBNP levels, peak oxygen uptake, or exercise duration compared to 60 bpm. However, we found a significant drop in sympathetic activity, as measured by bursts/100 RR, in the 80-bpm study period, which represented a large decrease in the levels of sympathetic activity, as measured by the effect size. Yet, we failed to demonstrate any significant decrease in bursts/min. Interestingly, the patients reported higher scores (indicating less limitation) in the QoL surveys in some health dimensions during the 60-bpm study period. To the best of our knowledge, this study is the first to conduct a series of direct measurements of the sympathetic nervous system in CRT patients, with varying atrial pacing rates in an intermediate 3-month time period.

Ståhlberg et al. observed that short-term increased atrial pacing (80 bpm vs. 60 bpm), significantly decreases the average estimated pulmonary artery diastolic, right ventricular systolic and diastolic pressures, and increases cardiac output. However, the same parameters appeared to increase during the 2-week, 80-bpm period, raising concerns for the long-term effects of HR elevation. Furthermore, in accord with our study, no significant changes were observed in the BNP levels or in the 6-minute walk test results between the 80-bpm and 60-bpm study periods. [8] The absence of clinical effects may be attributed to subtle changes that occur but cannot be detected by usual clinical tests. In a newly published paper, by the same author group, on the acute effects of increasing CRT pacing rates on hemodynamic control and MSNA a very similar combination of changes were reported. During 10 minutes of increased pacing rates (from 50 to 90-bpm) a decreased MSNA burst incidence of 44% was reported (about 35% decrease with the step from 70 to 90-bpm). [33]

Recently, in an elegantly designed trial (PEGASUS) [34], 1,309 heart failure patients with newly implanted CRT devices were randomized to a DDD-40, DDDR-40 or DDD-70 group and followed for 10.5 months. No significant differences were observed in the composite clinical endpoint of all-cause mortality, HF events, NYHA functional class, or patient global self-assessments between the different basal pacing rates. A direct comparison between our study and PEGASUS is difficult because of dissimilar endpoints, and data regarding BNP and working capacity (pVO_2_) is yet to be published from PEGASUS. However, in contrast to our study, the mean HR difference in the PEGASUS trial was only 2–3 bpm. Moreover, the percent atrial pacing was only 43% during DDD-70, while it was greater than 82% during our 80-bpm study period. It could be speculated that a greater difference in HR and atrial pacing, and perhaps a longer follow-up period, are needed to generate a difference in clinical outcome.

It has been well established that elevated sympathetic activity is typical [30] and is well correlated to symptoms, progression and poor prognosis for heart failure,[35, 36] and elevated values have been associated with heart failure mortality by Barretto et al. [30] Previously, Kuniyoshi et al. [37] and Hamdan et al. [38] have demonstrated, using MSNA, that sympathetic activity decreases after CRT device implantation. With the lower sympathetic tone at 80 bpm, our data suggest that a further increase in the basal atrial pacing rate could prove to be beneficial for heart failure patients undergoing CRT therapy.

The exact mechanisms behind the lower sympathetic tone during a higher atrial basal pacing rate are not known. Nonetheless, results from previous short-term biventricular pacing studies [39, 40] indicate that an arterial baroreceptor-reflex-mediated sympathoinhibition is associated with higher pacing rates. This finding is partly derived from the decreased pulmonary arterial resistance and total peripheral resistance, thus facilitating an afterload reduction. We did not encounter any changes in blood pressure, which could indicate that any changes were subtle and not measurable by our equipment and could indicate a high risk of statistical type II error due to the small number of patients. Other studies have confirmed that reducing cardiac filling pressure lowers norepinephrine spill over [41] in HF patients, which is then mirrored by decreased sympathetic activity, as measured by MSNA. [42] Thus, the diminished sympathetic activity during the 80-bpm study period could also be the result of lower cardiac filling pressure. A decrease in cardiac filling pressures would be expected to result in lower NT-proBNP values, which was not observed in the current study, thereby questioning lower filling pressures as an explanation for the decrease in MSNA.

DeMazumder et al. recently demonstrated that cholinergic signaling is highly up-regulated in the failing LV, and moreover, differential remodeling of cholinergic signaling is important for the return of sympathovagal stability by CRT. [43] Whether this novel mechanism can influence muscle sympathetic activity is unknown, but nonetheless suggests that future studies evaluating CRT treatment should implement parasympathetic measurements.

We emphasize that we did not achieve significant difference between the two study periods in burst frequency (MSNA per minute). However, this was anticipated due to manipulation of HR and the fact that MSNA is strictly linked to the cardiac cycle by the arterial baroreflex. The incidence of MSNA bursts is strongly related to the afferent input from the baroreceptors. Thus, sympathetic outflow from the brainstem only occurs in conjunction with diastole and only with those diastoles, which do not activate baroreceptors. This has led to the prevailing view that MSNA burst incidence is an indication of the level of central sympathetic outflow. Burst frequency is usually considered to illustrate the degree of activity reaching the effector organs, whereas Burst incidence reflects central control in the sense that it illustrates the relative amount of cardiac cycles filled with MSNA bursts [44–46]

Another major concern is falling stroke volumes (SV) with elevated pacing rates. However, the hemodynamic changes suggest that cardiac output is maintained and, in some instances, increased (likely due to increased contractility and decreased filling pressures). These mechanisms seem to be in place up to at least 100–120 bpm, whereas there is a shift toward sympathoexcitation at higher pacing rates. [39, 40]

BNP is a valuable parameter for monitoring the effects of CRT in HF patients,[47] and prior studies have shown the importance of post-CRT BNP levels in predicting HF events and death. [48, 49] Furthermore, Brenyo et al. established that the pattern of BNP change from baseline to 1 year is an independent predictor of subsequent clinical response. [49] We did not encounter a significant increase in BNP levels during the two study periods, which indicates that a basal rate of 80 bpm is not harmful. However, the long-term effects of a higher basal rate beyond three months are unknown.

It was anticipated that many of our patients would not achieve a RER>1.1 (an indicator of an adequately performed test), because evidence suggests that nearly 50% of HF patients are unable to reach this target. [22] Recent data suggest that results below the target RER do not invalidate the key parameters of pVO_2_ and the pVE/CO_2_ slope; in contrast, these measurements remain as strong prognostic variables, irrespective of RER. Furthermore, the pVE/CO_2_ slope is considered to be an effort-independent variable and seems to be superior to pVO_2_ as a prognostic variable. [22] We observed an increase in pVO_2_ and a decrease in the pVE/CO_2_ slope from 60 bpm to 80 bpm; however, these observations were marginal and not significant. The measured range of pVO_2_ in our study is comparable to those of previous studies [50] performed in similar patient populations. Furthermore, the baseline values of pVO_2_ and of the pVE/CO_2_ slope indicate improved exercise tolerance in our study; thus, it is not surprising that exercise capacity did not improve. Likewise, larger trials [51, 52] conducted in CRT patients with NYHA II, with integrated power to detect changes in exercise capacity (pVO_2_, workload, etc.), have also been unable to demonstrate a significant improvement in these parameters despite clear improvements in cardiac structure and function. Additionally, a follow-up period of 3 months may not be sufficient to detect exercise improvement. However, the lack of statistical power cannot be ruled out as an explanation for the absence of a significant increase in pVO_2_ or decrease in the pVE/CO_2_ slope.

The LIfHE questionnaire did not reveal any differences between the 60- and 80-bpm study periods, thus indicating no adverse or beneficial effect of increased atrial pacing on QoL. As stated earlier, SF-36 is comprised of 36 items, which are subdivided into 8 health dimensions. [20] The study population reported higher (better) scores in only two of the 8 health dimensions during the 60-bmp compared to the 80-bmp study period: “bodily pain” and “component score of mental health”. This result is surprising because no verbal notification of increased bodily pain or lower mental health were conveyed during or after the 80-bpm period examinations. Moreover, no patient reported any discomfort that resulted in premature discontinuation during the 80-bpm period, and the calculated effect sizes revealed only a medium-level change in these parameters. One possible explanation for less “bodily pain” at 60 bpm could be a lower sensation of palpitation and a frequency closer to the physiological level. In addition, constantly increased HRs might manifest as enhanced bodily excitement/tension/stress and irritation, which might result in lower “mental health” comfort. A recent meta-analysis revealed that patients with NYHA I-II did not enjoy the same improvements in QoL as patients with NYHA III-IV when comparing CRT only with an implantable cardioverter defibrillator (ICD). [19] The majority of our patients were in NYHA class II, and perhaps they already had relatively high baseline scores that were offset by manipulating their pacemaker settings.

## Limitations

This pilot study has several limitations. First, we only obtained a modest increase in the mean HR (i.e., 9 bpm) during the “fast” pacing period. This result was a consequence of a relatively high intrinsic HR, which increased the mean HR during DDD-60 pacing. One may think that more separation in the HR measurements would have resulted in a more pronounced difference between the two intervals.

Second, our data are based on constant AV delays and might not apply to different AV delays. However, Scharf et al. demonstrated that longer AV delays, unlike traditional pacemakers, were required to obtain optimal cardiac output with increasing HR when using CRT devices. Thus, we may have observed greater decreased sympathetic activity with dynamic or prolonged AV delays. However, a prolonged AV-delay carries the risk of loss of BiV pacing due to intrinsic conduction. Third, this study lacked norepinephrine measurements; however, a strong correlation with MSNA exists. [53] MSNA provides a more direct and accurate measurement of central sympathetic activity compared to catecholamine dosage or HR variability (not possible to perform due to dependence on intrinsic and normal sinus rhythm). In addition, the small number of participants in this study prevents firm conclusions, particularly when no differences were observed; however, the prospective randomized crossover design and 3-month follow-up provides greater validity and power to this study. Despite the intention to blind patients to their pacing rates, the possibility of detecting the actual pacing rate by sensation or home monitoring cannot be excluded. Moreover, our results apply only to the rates of 60 bpm and 80 bpm and cannot simply be extracted to higher basal atrial pacing rates. Furthermore, no NYHA IV patients were included in this study. Therefore, the results may not be applicable to populations with more severe disease. Yet, with the expansion of CRT to patients with mild HF,[51] this study is still relevant.

## Conclusion

A modest increase in the atrial pacing rate for 3 months in non-ischemic CRT patients appears to decrease sympathetic tone (burst incidence only), as directly measured by MSNA. However, Qol (SF-36) revealed less “bodily pain” and better “mental health” during the DDD-60 study period, which is of clinical importance.

These findings may have possible implications for clinical decision-making when programming CRT devices.

Further studies are needed to determine whether increased atrial pacing leads to better long-term clinical outcomes and survival in patients with mild HF symptoms and whether the decreased sympathetic activity benefits seen at 3 months are also present in the long term.

## Supporting Information

S1 CONSORT Checklist CONSORT checklist. (DOC)

S1 Protocol Protocol in original language. (DOC)

## References

[1] Cleland JG, Freemantle N, Erdmann E, Gras D, Kappenberger L, Tavazzi L, et al (2012) Long-term mortality with cardiac resynchronization therapy in the Cardiac Resynchronization-Heart Failure (CARE-HF) trial. Eur J Heart Fail 14: 628–634. 10.1093/eurjhf/hfs055 22552183

[2] Bristow MR, Saxon LA, Boehmer J, Krueger S, Kass DA, De Marco T, et al (2004) Cardiac-resynchronization therapy with or without an implantable defibrillator in advanced chronic heart failure. N Engl J Med 350: 2140–2150. 15152059 10.1056/NEJMoa032423

[3] Go AS, Mozaffarian D, Roger VL, Benjamin EJ, Berry JD, Borden WB, et al (2013) Heart disease and stroke statistics—2013 update: a report from the American Heart Association. Circulation 127: e6–e245. 10.1161/CIR.0b013e31828124ad 23239837 PMC5408511

[4] Farwell D, Patel NR, Hall A, Ralph S and Sulke AN (2000) How many people with heart failure are appropriate for biventricular resynchronization? Eur Heart J 21: 1246–1250. 10924314 10.1053/euhj.1999.1985

[5] Cuoco FA and Gold MR (2012) Optimization of cardiac resynchronization therapy: importance of programmed parameters. J Cardiovasc Electrophysiol 23: 110–118. 10.1111/j.1540-8167.2011.02235.x 22188487

[6] Gold MR, Niazi I, Giudici M, Leman RB, Sturdivant JL, Kim MH, et al (2011) A prospective, randomized comparison of the acute hemodynamic effects of biventricular and left ventricular pacing with cardiac resynchronization therapy. Heart Rhythm 8: 685–691. 10.1016/j.hrthm.2010.12.039 21193063

[7] Jansen AH, Bracke FA, van Dantzig JM, Meijer A, van der Voort PH, Aarnoudse W, et al (2006) Correlation of echo-Doppler optimization of atrioventricular delay in cardiac resynchronization therapy with invasive hemodynamics in patients with heart failure secondary to ischemic or idiopathic dilated cardiomyopathy. Am J Cardiol 97: 552–557. 16461055 10.1016/j.amjcard.2005.08.076

[8] Stahlberg M, Hilpisch K, Reiters P, Linde C and Braunschweig F (2013) Haemodynamic effects of different basic heart rates in ambulatory heart failure patients treated with cardiac resynchronization therapy. Europace 15: 1182–1190. 10.1093/europace/eus423 23277532

[9] Stahlberg M, Lund LH, Zabarovskaja S, Gadler F, Braunschweig F and Linde C (2012) Cardiac resynchronization therapy: a breakthrough in heart failure management. J Intern Med 272: 330–343. 10.1111/j.1365-2796.2012.02580.x 22882554

[10] Voss F, Becker R, Hauck M, Katus HA and Bauer A (2009) The basic pacing rate in CRT patients: the higher the better? Clin Res Cardiol 98: 219–223. 10.1007/s00392-009-0745-2 19219396

[11] Tse HF, Siu CW, Lee KL, Fan K, Chan HW, Tang MO, et al (2005) The incremental benefit of rate-adaptive pacing on exercise performance during cardiac resynchronization therapy. J Am Coll Cardiol 46: 2292–2297. 16360061 10.1016/j.jacc.2005.02.097

[12] Sims DB, Mignatti A, Colombo PC, Uriel N, Garcia LI, Ehlert FA, et al (2011) Rate responsive pacing using cardiac resynchronization therapy in patients with chronotropic incompetence and chronic heart failure. Europace 13: 1459–1463. 10.1093/europace/eur127 21551475

[13] Brubaker PH and Kitzman DW (2007) Prevalence and management of chronotropic incompetence in heart failure. Curr Cardiol Rep 9: 229–235. 17470336 10.1007/BF02938355

[14] Dresing TJ, Blackstone EH, Pashkow FJ, Snader CE, Marwick TH and Lauer MS (2000) Usefulness of impaired chronotropic response to exercise as a predictor of mortality, independent of the severity of coronary artery disease. Am J Cardiol 86: 602–609. 10980208 10.1016/s0002-9149(00)01036-5

[15] Voigt A, Shalaby A, Adelstein E and Saba S (2010) Beta-blocker utilization and outcomes in patients receiving cardiac resynchronization therapy. Clin Cardiol 33: E1–5. 10.1002/clc.20500 PMC6653697 20549778

[16] Bohm M, Swedberg K, Komajda M, Borer JS, Ford I, Dubost-Brama A, et al (2010) Heart rate as a risk factor in chronic heart failure (SHIFT): the association between heart rate and outcomes in a randomised placebo-controlled trial. Lancet 376: 886–894. 10.1016/S0140-6736(10)61259-7 20801495

[17] Wilkoff BL (2003) The Dual Chamber and VVI Implantable Defibrillator (DAVID) Trial: rationale, design, results, clinical implications and lessons for future trials. Card Electrophysiol Rev 7: 468–472. 15071277 10.1023/B:CEPR.0000023165.20987.b1

[18] Brignole M, Auricchio A, Baron-Esquivias G, Bordachar P, Boriani G, Breithardt OA, et al (2013) 2013 ESC guidelines on cardiac pacing and cardiac resynchronization therapy: the task force on cardiac pacing and resynchronization therapy of the European Society of Cardiology (ESC). Developed in collaboration with the European Heart Rhythm Association (EHRA). Europace 15: 1070–1118. 10.1093/europace/eut206 23801827

[19] Chen S, Yin Y and Krucoff MW (2012) Effect of cardiac resynchronization therapy and implantable cardioverter defibrillator on quality of life in patients with heart failure: a meta-analysis. Europace 14: 1602–1607. 10.1093/europace/eus168 23104857

[20] Bjorner JB, Damsgaard MT, Watt T and Groenvold M (1998) Tests of Data Quality, Scaling Assumptions, and Reliability of the Danish SF-36. J Clin Epidemiol 51: 1001–1011. 9817118 10.1016/s0895-4356(98)00092-4

[21] Rector TS and Cohn JN (1992) Assessment of patient outcome with the Minnesota Living with Heart Failure questionnaire: Reliability and validity during a randomized, double-blind, placebo-controlled trial of pimobendan. Am Heart J 124: 1017–1025. 1529875 10.1016/0002-8703(92)90986-6

[22] Chase PJ, Kenjale A, Cahalin LP, Arena R, Davis PG, Myers J, et al (2013) Effects of respiratory exchange ratio on the prognostic value of peak oxygen consumption and ventilatory efficiency in patients with systolic heart failure. JACC Heart Fail 1: 427–432. 10.1016/j.jchf.2013.05.008 24621975 PMC7296992

[23] Edgell H and Stickland MK (2014) Activation of the carotid chemoreflex secondary to muscle metaboreflex stimulation in men. Am J Physiol Regul Integr Comp Physiol 306: R693–700. 10.1152/ajpregu.00472.2013 24573180

[24] Evangelista A, Flachskampf F, Lancellotti P, Badano L, Aguilar R, Monaghan M, et al (2008) European Association of Echocardiography recommendations for standardization of performance, digital storage and reporting of echocardiographic studies. Eur J Echocardiogr 9: 438–448. 10.1093/ejechocard/jen174 18579482

[25] Wellek S and Blettner M (2012) On the proper use of the crossover design in clinical trials: part 18 of a series on evaluation of scientific publications. Dtsch Arztebl Int 109: 276–281. 10.3238/arztebl.2012.0276 22567063 PMC3345345

[26] Moreadith CW, Sollecito W and Koch G (1986) Analysis of crossover studies with multiple baseline measurements. Controlled Clinical Trials 7: 253.

[27] Kenward MG and Roger JH (2010) The use of baseline covariates in crossover studies. Biostatistics 11: 1–17. 10.1093/biostatistics/kxp046 19915170

[28] Fleiss JL, Wallenstein S and Rosenfeld R (1985) Adjusting for baseline measurements in the two-period crossover study: A cautionary note. Controlled Clinical Trials 6: 192–197. 4042663 10.1016/0197-2456(85)90002-9

[29] Fritz CO, Morris PE and Richler JJ (2012) Effect size estimates: current use, calculations, and interpretation. J Exp Psychol Gen 141: 2–18. 10.1037/a0024338 21823805

[30] Barretto AC, Santos AC, Munhoz R, Rondon MU, Franco FG, Trombetta IC, et al (2009) Increased muscle sympathetic nerve activity predicts mortality in heart failure patients. Int J Cardiol 135: 302–307. 10.1016/j.ijcard.2008.03.056 18582965

[31] Najem B, Unger P, Preumont N, Jansens JL, Houssiere A, Pathak A, et al (2006) Sympathetic control after cardiac resynchronization therapy: responders versus nonresponders. Am J Physiol Heart Circ Physiol 291: H2647–2652. 16844919 10.1152/ajpheart.00373.2006

[32] Altman DG. Practical Statistics for Medical Research. London: Chapman & Hall; 1990.

[33] Stahlberg M, Sander M, Mortensen L, Linde C and Braunschweig F (2015) Increase in paced heart rate reduces muscle sympathetic nerve activity in heart failure patients treated with cardiac resynchronization therapy. Europace 17: 439–446. 10.1093/europace/euu289 25355780

[34] Martin DO, Day JD, Lai PY, Murphy AL, Nayak HM, Villareal RP, et al (2012) Atrial support pacing in heart failure: results from the multicenter PEGASUS CRT trial. J Cardiovasc Electrophysiol 23: 1317–1325. 10.1111/j.1540-8167.2012.02402.x 22830441

[35] Esler M and Kaye D (2000) Measurement of sympathetic nervous system activity in heart failure: the role of norepinephrine kinetics. Heart Fail Rev 5: 17–25. 16228913 10.1023/A:1009889922985

[36] Grassi G, Bolla G, Quarti-Trevano F, Arenare F, Brambilla G and Mancia G (2008) Sympathetic activation in congestive heart failure: reproducibility of neuroadrenergic markers. Eur J Heart Fail 10: 1186–1191. 10.1016/j.ejheart.2008.09.013 18851926

[37] Kuniyoshi RR, Martinelli M, Negrao CE, Siqueira SF, Rondon MU, Trombetta IC, et al (2013) Effects of Cardiac Resynchronization Therapy on Muscle Sympathetic Nerve Activity. Pacing Clin Electrophysiol. 10.1111/pace.12254 23952584

[38] Hamdan MH, Barbera S, Kowal RC, Page RL, Ramaswamy K, Joglar JA, et al (2002) Effects of resynchronization therapy on sympathetic activity in patients with depressed ejection fraction and intraventricular conduction delay due to ischemic or idiopathic dilated cardiomyopathy. Am J Cardiol 89: 1047–1051. 11988193 10.1016/s0002-9149(02)02273-7

[39] Stahlberg M, Kessels R, Linde C and Braunschweig F (2011) Acute haemodynamic effects of increase in paced heart rate in heart failure patients recorded with an implantable haemodynamic monitor. Europace 13: 237–243. 10.1093/europace/euq354 20952424

[40] Segerson NM, Wasmund SL, Daccarett M, Fabela ML, Hammond CH, Stoddard G, et al (2008) The acute effect of atrioventricular pacing on sympathetic nerve activity in patients with normal and depressed left ventricular function. Am J Physiol Heart Circ Physiol 295: H1076–H1080. 10.1152/ajpheart.91404.2007 18586896 PMC2544507

[41] Azevedo ER, Newton GE, Floras JS and Parker JD (2000) Reducing cardiac filling pressure lowers norepinephrine spillover in patients with chronic heart failure. Circulation 101: 2053–2059. 10790346 10.1161/01.cir.101.17.2053

[42] Floras JS (2009) Sympathetic nervous system activation in human heart failure: clinical implications of an updated model. J Am Coll Cardiol 54: 375–385. 19628111 10.1016/j.jacc.2009.03.061

[43] DeMazumder D, Kass DA, O'Rourke B and Tomaselli GF (2015) Cardiac resynchronization therapy restores sympathovagal balance in the failing heart by differential remodeling of cholinergic signaling. Circ Res 116: 1691–1699. 10.1161/CIRCRESAHA.116.305268 25733594 PMC4577523

[44] Sundlof G and Wallin BG (1978) Muscle-nerve sympathetic activity in man. Relationship to blood pressure in resting normo- and hyper-tensive subjects. Clin Sci Mol Med Suppl 4: 387s–389s. 282094 10.1042/cs055387s

[45] Kramer HH, Ament SJ, Breimhorst M, Klega A, Schmieg K, Endres C, et al (2014) Central correlation of muscle sympathetic nerve activation during baroreflex unloading—a microneurography-positron emission tomography study. Eur J Neurosci 39: 623–629. 10.1111/ejn.12437 24528135

[46] Sundlof G and Wallin BG (1977) The variability of muscle nerve sympathetic activity in resting recumbent man. J Physiol 272: 383–397. 592196 10.1113/jphysiol.1977.sp012050 PMC1353564

[47] Wang RX, Guo T and Li XR (2009) BNP/NT-proBNP and cardiac pacing: a review. Pacing Clin Electrophysiol 32: 794–799. 10.1111/j.1540-8159.2009.02369.x 19545345

[48] Berger R, Shankar A, Fruhwald F, Fahrleitner-Pammer A, Freemantle N, Tavazzi L, et al (2009) Relationships between cardiac resynchronization therapy and N-terminal pro-brain natriuretic peptide in patients with heart failure and markers of cardiac dyssynchrony: an analysis from the Cardiac Resynchronization in Heart Failure (CARE-HF) study. Eur Heart J 30: 2109–2116. 10.1093/eurheartj/ehp210 19493864

[49] Brenyo A, Barsheshet A, Rao M, Huang DT, Zareba W, McNitt S, et al (2013) Brain natriuretic peptide and cardiac resynchronization therapy in patients with mildly symptomatic heart failure. Circ Heart Fail 6: 998–1004. 10.1161/CIRCHEARTFAILURE.112.000174 23801020

[50] Auricchio A, Kloss M, Trautmann SI, Rodner S and Klein H (2002) Exercise performance following cardiac resynchronization therapy in patients with heart failure and ventricular conduction delay. Am J Cardiol 89: 198–203. 11792342 10.1016/s0002-9149(01)02200-7

[51] Abraham WT, Young JB, Leon AR, Adler S, Bank AJ, Hall SA, et al (2004) Effects of cardiac resynchronization on disease progression in patients with left ventricular systolic dysfunction, an indication for an implantable cardioverter-defibrillator, and mildly symptomatic chronic heart failure. Circulation 110: 2864–2868. 15505095 10.1161/01.CIR.0000146336.92331.D1

[52] Higgins SL, Hummel JD, Niazi IK, Giudici MC, Worley SJ, Saxon LA, et al (2003) Cardiac resynchronization therapy for the treatment of heart failure in patients with intraventricular conduction delay and malignant ventricular tachyarrhythmias. J Am Coll Cardiol 42: 1454–1459. 14563591 10.1016/s0735-1097(03)01042-8

[53] Lambert EA, Schlaich MP, Dawood T, Sari C, Chopra R, Barton DA, et al (2011) Single-unit muscle sympathetic nervous activity and its relation to cardiac noradrenaline spillover. J Physiol 589: 2597–2605. 10.1113/jphysiol.2011.205351 21486790 PMC3115828

