# Supplementary material for: Optimal Cardiac Resynchronization Therapy Pacing Rate in Non-Ischemic Heart Failure Patients: A Randomized Crossover Pilot Trial
Source: PLoS One. 2015 Sep 18;10(9):e0138124. doi: 10.1371/journal.pone.0138124 (PMC4575161; doi:10.1371/journal.pone.0138124)
Supplement: S1 Protocol — (DOC) [file pone.0138124.s002.doc]

6. marts 2011

**Optimal hjertefrekvens under Cardiac resynchronization therapy (CRT)**

**Projektbeskrivelse**

**Projektansvarlige**

Adam Ali Ghotbi, læge

Lars Køber, professor, overlæge, dr. med.

Finn Gustafsson, overlæge, dr. med.

Regitze Videbæk, overlæge

Helen Høgh Petersen, overlæge

Berit Thornvig, afdelingslæge, ph.d.

Jesper Hastrup-Svendsen, professor, overlæge, dr. med.

Peter Karl Jacobsen, 1. reservelæge dr.med.

**Institution**

Rigshospitalet, Hjertecentret afd. B

Blegdamsvej 9

2100 Kbh. Ø

**Formål**

At belyse hvilken hjertefrekvens, der er optimal for CRT behandling hos patienter med iskæmisk og non-iskæmisk hjertesvigt.

**Baggrund**

En del af patienter med hjertesvigt har udover reduceret pumpekraft af venstre hjertehalvdel også ændret funktion af hjertets elektriske system. Typisk ses for langsom udbredelse af elektriske signaler i hjertets hovedkamre (såkaldt grenblok). Dette medfører, at den normale synkroniserede sammentrækning af hjertet forstyrres og pumpekraften falder yderligere pga. dyssynkroni.

Cardiac resynchronization therapy (CRT) er en type pacemaker behandling som har til formål at reetablere synkroniseringen og dermed øge hjertets pumpekraft. Dette gøres ved at pace flere steder samtidig og dermed kompensere for det syge hjertes langsomme elektriske ledningsevne. Den gavnlige effekt af CRT forudsætter, at CRT pacemakeren pacer så tæt på 100% af tiden som muligt. CRT er en effektiv behandling for patienter med behandlings refraktær hjertesvigt og elektrisk dyssynkroni[[1]](#endnote-2). Flere studier har vist signifikant reduktion i mortalitet og morbiditet i hjertesvigts patienter efter CRT implantation[[2]](#endnote-3),[[3]](#endnote-4),[[4]](#endnote-5).

CRT behandlingen har konsekvent medført bedring af symptomer, livskvalitet og arbejdskapacitet hos patienter med nedsat venstre ventrikelfunktion (defineret som LVEF < 35%) og elektrisk dyssynkroni (grenblok)[[5]](#endnote-6) .

CRT’s gavnlige effekt på blandt andet færre hospitals indlæggelser og mortalitet tilskrives til dels en forbedring i venstre ventrikels (LV) geometri, nemlig mindsket LV volumen og dimensioner og øget pumpefunktion (LVEF)[[6]](#endnote-7).

Der foreligger ikke undersøgelse af hvad den optimale hjertefrekvens er under CRT behandling. På den ene side viser det sig at patienter, som undergår CRT behandling og samtidig får medicinsk behandling med betablokkere (som sænker hjertefrekvensen) har en bedre overlevelse og morbiditet i forhold til CRT patienter uden betablokker behandling[[7]](#endnote-8). Dette er i tråd med andre studier, som påviser hjertefrekvensnedsættelses gode effekt på mortalitet og morbiditet hos patienter med hjertesvigt, dog uden CRT behandling[[8]](#endnote-9).

På den anden siden tyder flere mindre studier på, at højere hjertefrekvens under CRT terapi kan være gavnligt. Disse studier viser øget minutvolumen i takt med stigende biventrikulær hjertefrekvens[[9]](#endnote-10). Ligeledes tyder korttidsstudier på faldende sympatikus aktivitet ved højere pacefrekvens under CRT terapi.[[10]](#endnote-11) Årsagen henføres til reduceret hjerte fyldningstryk og vaskulær modstand samtidig med øget minutvolumen og diastolisk tryk. Det sidste medfører refleks sympatikus inhibition via barorecptorer[[11]](#endnote-12).

I dette studie vil vi søge, at belyse hvilken pacefrekvens/hjertefrekvens der er den optimale hos patienter med CRT behandling. Dette gøres ved at sammenligne en basalpacefrekvens/ hjertefrekvens på 60 og 80 slag /min. Dette vil medføre en gennemsnitlig forskel i hjertefrekvens på ca. 15 slag/min gennem hele døgnet. Der vurderes ændringer i kliniske, ekkokardiografiske og biokemiske variable, såsom NT-pro BNP samt sympatikus aktivitet.

**Statistiske overvejelser**

Det primære endepunkt er en kontinuert variable (NT-proBNP) som sammenlignes mellem matchede par (patienten er sin egen control). Det forventes at NT-proBNP ikke er normalfordelt, hvorfor denne logaritmiseres. Tidligere undersøgelser viser standard deviationen af ændringer på 100 pg/ml og en relevant forskel mellem behandlingerne vil være 50 pg/ml. Med en power på 80% og en type I fejl sat til 5% vil en nulhypotese kunne afvises med en størrelse af stikprøven på 33 personer. Størrelsen af inkluderede patienter sættes til 40 for at have plads til at 7 udgår før tid.

**Studiedesign**

Randomized single blinded prospektivt cross -over studie.

**Materiale og Metode**

40 patienter med stabil iskæmisk og non-iskæmisk hjertesvigt og biventrikulær pacemaker enhed (CRT) randomiseres (dobbelt) blindet til i tilfældig rækkefølge at paces med basalrate på 60 BPM i 3 mdr. og 80 BPM i 3 mdr. Patienterne skal på inklusionstidspunktet være hæmodynamisk stabile, LVEF < 35 % og haft CRT i mindst 6 måneder og paces i begge hovedkamre (BIV paces) > 90% af tiden. Alder over 18 år.

Eksklusionskriterier: Svær komorbiditet, kronisk obstruktiv lungelidelse, kræft, Plasma Kreatinin > 200 mikromol/L.

CRT patienter i NYHA I.

Der planlægges ikke ændringer i patienternes vanlige hjertesvigtsmedicin, men diuretika kan frit justeres i løbet af forsøget.

Der foretages kontrol af 3 omgange – baseline, og ved afslutning af hver behandlingsperiode, dvs. 0, 3 og 6 mdr.

Patienterne møder på Rigshospitalet i pacemaker ambulatoriet. CRT enheden sættes i tilfældig rækkefølge til basal pace rate på 60 BPM i den ene periode og i den anden periode øges basal pace rate til 80 BPM. Af hensyn til blindingen vil to bioanalytikere knyttes til denne funktion.

Endpoints:

- Primært endepunkt: NT-pro BNP. Blodprøve af 40 ml per gang. Der oprettes ikke Biobank.
- Vo2 Max
- Ekkokardiografi med fokus på venstre ventrikel: LVEDD/LVESD, LVEF, CO, SV
- Diuretika dosis-ændringer
- Ventrikulær aktivitet (VES og VT) + Atrieflimren byrde
- CRT enhedens registrering af fysisk aktivitets niveau
- CRT enhedens registrering af hjertefrekvens variabilitet
- Sympatikus aktivitet
- Spørgeskema mhp symptomstatus

**Sikkerhedshensyn**

Studiet stoppes hvis flere end 5 patienter stiger > 50% i NT-proBNP sammenlignet med baseline eller stiger > 1 NYHA klasse under ”hurtig pace” uden anden oplagt årsag end ændringer i  CRT indstillinger. Der tilknyttes en sikkerhedsmonitor med adgang til behandlingskoden. Dvs. alle ændringer i NT-proBNP > 50% eller NYHA > 1 vurderes af sikkerhedsmonitor, som sørger for disse patienter udgår af studiet og pacemaker funktionen omprogrammeres til udgangspositionen . Ved mistanke om klinisk forværring tages ekstra NT-proBNP.

**Etiske overvejelser**

Det er vigtigt at få belyst den optimale indstilling af CRT pacemaker. Herved kan patienter i CRT pacemaker behandling få det bedst mulige gavn af apparatet. Der er på nuværende tidspunkt ikke nogle retningslinjer for hvilken hjertefrekvens der er den bedste. I forhold til eventuelle risici og bivirkninger (se næste afsnit) er fordelene store ved indgåelse i studiet. For den enkelte forsøgsdeltager medfører studiet en stor klinisk kontrol, fx hjerteskanning, blodprøver, spørgeskemaer og fysisk formåen. Desuden afdækkes forsøgsdeltagerens bedst mulige gavn af CRT pacemakeren. Ved forsøgets afslutning vil der være videnskabelige beviser for, hvilken pacefrekvens der er mest optimal for CRT patienter generelt.

Det er rutine i den daglige klinik at øge pacefrekvensen hvis man står i situationer hvor CRT enheden skal optimeres, f.eks. ved atrieflimren (forkammer flimren).

**Risici og Bivirkninger**

Forsøgsdeltagerne vil måske kunne mærke den øgede pacefrekvens i begyndelsen. Dette vil for de fleste ikke føles ubehageligt, men nogle vil kunne mærke forbigående fornemmelse af let hjertebanken, hovedpine og svimmelhed. Såfremt disse persisterer kan deltagerne udgå af studiet.

Mere alvorligere risici vurderes at være usandsynlige, men hvis der opstår forværring i hjertesvigt, åndenød eller brystsmerter (angina pectoris) udgår patienten (jf. ovenfor). Øvrige former for gener er ubehag i forbindelse med blodprøvetagning og nerveaktivitets måling i form af ømhed / hæmatom.

Samlet tidsforbrug for den enkelte deltager vurderes til 3 dage med kontrol inkl. om programmering af pacemaker i løbet af 3 mdr. De fleste patienter undgår en planlagt ambulant kontrol i løbet af studiet.

**Datatilsynet**

Projektet er meldt til Datatilsynet

**Tidsplan**

Afventer etisk videnskabelig komite

Marts 2011 - april 2011 indsamling af patient population og praktisk forberedelse

Medio maj 2011 opstart af studie med første patienter

Medio september 2011 første cross-over og del resultater

Medio oktober 2011 afslutning af første hold patienter

Oktober 2011 – december 2011 indsamling af data

December 2011 – januar 2012 bearbejdning af date og skrivning

Resultater forventes klar medio april 2012

**Økonomiske forhold**

Den forsøgsansvarlige modtager ikke vederlag for gennemførelsen af forsøget. Initiativtagerne bag forsøget er Peter Karl Jacobsen, 1. reservelæge dr.med., Adam Ali Ghotbi, læge, Lars Køber, professor, overlæge, dr. med. De forsøgsansvarlige er ikke økonomisk tilknyttet private virksomheder. Der vil blive behov for midler til dækning af læge løn og til øvrige udgifter i forbindelse med projektet i forbindelse med bearbejdning af data.

**Retningslinjer for indhentelse af informeret samtykke**

Potentielle deltagere modtager først et brev indeholdende information omkring studiet. Såfremt accept af deltager arrangeres et telefonisk kontakt af de projektansvarlige mhp. aftale om mødetidspunkt for yderligere mundtlig information ved en af de forsøgsansvarlige. Den mundtlige information foregår derved efter den skriftlige information/brev.

Forsøgsdeltagerne informeres om mulighed for at medbringe bisidder ved både det skriftlige information og det telefoniske kontakt.

Informationssamtalen vil foregå på Rigshospitalet i uforstyrret og trygge rammer. Samtykket søges indhentet efter den mundtlige information. Der vil være mulighed for betænkningstid efter såvel brev/skriftlig information af mindst 1 uges varighed samt efter mundtlig information af mindst 1 time.

**Offentliggørelse af data/publikation**

Studiet vil blive offentliggjort i et anerkendt internationalt medicinsk tidsskrift eller kongres eller på Rigshospitalets hjemmeside.

Udover de projektansvarlige har øvrige medhjælpere ret til medforfatterskab Hovedforfattere, dvs. første og sidste forfattere, vil være Adam Ali Ghotbi og Lars Køber samt Peter Karl Jacobsen

**Reference**

1. Van Bommel RJ, Delagdo V, Schalij MJ et al. Critical Appraisal of the Use of Cardiac Resynchronization Therapy Beyond Current Guidelines. JACC 2010;754-62 [↑](#endnote-ref-2)
2. Abraham WT, Fisher WG, Smith AL et al. Cardiac Resynhronization in chronic heart failure. N Engl J Med 2002;346:1845-53 [↑](#endnote-ref-3)
3. Bristow MR, Saxon LA, Boehmer J et al. Cardiac Resynhronization therapy with or without an implantable defibrillator in advanced chronic heart failure. N Engl J Med 2004;44:1834-40 [↑](#endnote-ref-4)
4. Cleland JG, Daubert JC, Erdmann E, et al. Baseline characteristics of patients recruited into the CARE-HF study. Eur J Heart Fail

   2005;7:205-14. [↑](#endnote-ref-5)
5. Rivero-Ayerza M, Theuns D, Garcia-Garcia H el al. Eurpean Heart J 2006;27:2682-2688 [↑](#endnote-ref-6)
6. Solomon A, Foster E et al. Effect of cardiac resynchronization therapy on reverse remodeling and relation to outcome: multicenter automatic defibrillator implantation trial: cardiac resynchronization therapy. Circulation 2010; Sep 7;122(10):985-92 [↑](#endnote-ref-7)
7. Voight A, Shalaby A, Adelstein E et al. Beta-blocker Utilization and Outcomes in Patients Receiving CRT. Clin Cardiol 2010;33,7, E1-E5 [↑](#endnote-ref-8)
8. Böhm M, Swedberg K, Komajda M et al. Heart rate as a risk factor in chronic heart failure (SHIFT): the association between heart rate and outcomes in a randomised placebo-controlled trial. Lancet 2010 Sep 11;376(9744):886-94 [↑](#endnote-ref-9)
9. Voss F, Becker R et al. The basic pacing rate in CRT patients: the higher the better? Clin Res Cardiol. 2009 Apr;98(4):219-2 [↑](#endnote-ref-10)
10. Ståhlberg M, Kessels R, et al. Acute hemodynamic effects of increase in paced heart rate in heart failure patients recorded with an implantable hemodynamic monitor. Ph.D Thesis. Karolinske Instituttet, Sweden. 2010 p:43 study III. [↑](#endnote-ref-11)
11. Ståhlberg M. Hemodynamic Aspects of Biventricular Pacing in Heart Failure. Ph.D Thesis. Karolinske Instituttet, Sweden. 2010 p:37 [↑](#endnote-ref-12)
